# Effectiveness and safety of pulse oximetry in remote patient monitoring of patients with COVID-19

**DOI:** 10.1093/eurpub/ckac129.303

**Published:** 2022-10-25

**Authors:** A Alboksmaty, T Beaney, S Elkin, J Clarke, A Darzi, P Aylin, AL Neves

**Affiliations:** 1 Department of Primary Care and Public Health, Imperial College London, London, UK; 2 PSTRC, Imperial College London, London, UK

## Abstract

**Context:**

A surge of COVID cases globally is often portrayed as “very likely”, which overwhelms health systems and challenges their capacities. A mitigation strategy is seen by remotely monitoring COVID patients in out-of-hospital settings to determine the risk of deterioration.

**Description of the problem:**

We need an indicator to enable remote monitoring of COVID patients at home that can be measured by a handy tool; pulse oximetry which measures peripheral blood oxygen saturation (SpO2). Evidence shows that SpO2 is a reliable indicator of deterioration among COVID patients. The UK initiated a national programme (COVID Oximetry @ Home (CO@H)) to assess the theory. The concept can be potentially applied in other countries in various settings. As part of CO@H, we conducted a systematic review of the evidence on the safety and effectiveness of pulse oximetry in remote monitoring of COVID patients.

**Results:**

Our review confirms the safety and potential effectiveness of pulse oximetry in remote home monitoring among COVID patients. We identified 13 research projects involving 2,908 participants that assessed the proposed strategy. Evidence shows the need to monitor at-rest and post-exertional SpO2. At-rest SpO2 of ≤ 92% or a decrease of 5% or more in post-exertional SpO2 should indicate care escalation. The recommended method for measuring at-rest SpO2 is after 5-10 min of rest, and assessing post-exertional SpO2 is after conducting a 1-min sit-to-stand test. We could not find explicit evidence on the impact on health service use compared with other models of care.

**Lessons:**

Remote monitoring of COVID patients could alleviate the pressure on health systems and save hospital resources. Monitoring SpO2 by pulse oximetry can be widely applied, including in resource-limited settings, as the tool is affordable, reliable, and easy to use.

**Key messages:**

• Adopting relevant health technologies in remote patient monitoring is critical to combat the pandemic.

• Pulse oximetry is an affordable, easy to use and widely available tool to monitor patients with COVID-19 at home.

